# Looking back to look forward—the history of VAD laws in Australia and future law reform in the Australian territories

**DOI:** 10.1093/medlaw/fwad030

**Published:** 2023-09-11

**Authors:** Kerstin Braun

**Affiliations:** School of Law and Justice, University of Southern Queensland, Toowoomba, Australia

**Keywords:** Advocacy, Assisted dying, Australia, Law reform, Political support, Public support

## Abstract

Taking one’s own life or attempting to do so has long been decriminalised in Australia. Aiding, counselling, or inciting another person to kill him or herself, however, remains a criminal offence. Yet, all six Australian States have now introduced laws allowing assistance in dying under certain circumstances. This article traces the recent history of Voluntary Assisted Dying (VAD) laws in Australia. It examines the introduction of the world’s first assisted dying legislation in the Northern Territory in 1995 followed by the Federal Government’s 1997 deprivation of the Territories’ power to legislate on assisted dying invalidating said law. It further considers the fifty-seven failed Bills attempting to achieve law reform in this context in Australian jurisdictions between 1993 and 2017 with a view to identifying what factors may have contributed to the continuing lack of success. This article then outlines the rapid introduction of VAD laws in all six Australian States decriminalising VAD over the span of only 5 years. It ponders what may have changed to bring about this law reform. This article closes by contemplating potential future law reform in the Australian Territories, which have been reinstated with jurisdiction to legislate on VAD in December 2022.

## I. INTRODUCTION

Australia is a Commonwealth and comprises six States^1^ as well as two internal Territories with degrees of self-government.^2^ Prior to 2017, it was unlawful in all Australian jurisdictions to participate in Voluntary Assisted Dying (VAD).^3^ Assisting another person in ending their life or ending another person’s life, even upon their express request and with their consent, gave rise to criminal liability.

The legal situation changed in 2017 when Victoria first introduced the *Voluntary Assisted Dying Act* 2017 (Vic) (‘Vic VAD Act’), which commenced in 2019. What followed was the introduction of the 2019 Western Australian *Voluntary Assisted Dying Act 2019* (WA) (‘WA VAD Act’), which commenced in 2021, and the 2021 Tasmanian *End-of-Life Choices (Voluntary Assisted Dying) Act 2021* (Tas) (‘Tas VAD Act’), which commenced in 2022. Also in 2021, South Australia passed the *Voluntary Assisted Dying Act 2021* (SA) (‘SA VAD Act’) and Queensland the *Voluntary Assisted Dying Act 2021* (Qld) (Qld VAD Act), which commenced in January 2023. As the last Australian State, in 2022 New South Wales passed the *Voluntary Assisted Dying Act 2022* (NSW) (‘NSW VAD Act’) with an expected commencement date of late 2023. VAD remains currently unlawful in the Australian Capital Territory (ACT) and the Northern Territory (NT). It was not until December 2022 that the Federal Government reinstated the Territories with jurisdiction to legislate on VAD.^4^

The Vic, WA, SA, Qld, and NSW VAD Acts define VAD as ‘the administration of a voluntary assisted dying substance’ which also ‘includes steps reasonably related to’ such administration.^5^ VAD may occur through a medical practitioner who administers a lethal substance to a person to bring about their death, so-called practitioner administration,^6^ and through a person taking a prescribed lethal substance to cause their own death, so-called self-administration.^7^ The VAD Acts have decriminalised participation in assisted dying in the six States if participation occurs within the framework of the legislation.^8^

This article traces the recent history of VAD laws in Australia. In doing so it examines the introduction of the world’s first assisted dying legislation in the Northern Territory in 1995 followed by the Federal Government’s 1997 deprivation of the Territories’ powers to legislate on VAD invalidating said law. It further considers the fifty-seven failed Bills attempting law reform in this context in Australian jurisdictions between 1993 and 2017 with a view to exploring possible factors contributing to their enduring defeat. Against the backdrop of almost a quarter century of failed law-reform attempts, this article then outlines the rapid introduction of VAD laws in all six Australian States in only 5 years. The point of the exercise is to examine whether factors can be identified that have contributed to making VAD lawful in Australian States.

Considering the rapid VAD law reform movement in all Australian states, this article closes by contemplating the possible impact of this development on VAD law reform in two Australian Territories, the Northern Territory and the Australian Capital Territory.

## II. VAD LAW REFORM IN AUSTRALIA PRIOR TO 2017

Prior to 2017, aiding another in taking their own life or taking the life of another, even upon their request and with their consent, was a criminal offence in all Australian jurisdictions. This changed for a brief time when the Northern Territory introduced the world’s first assisted dying law in 1995. The law was invalidated when the Federal Government deprived the Territories of their power to legislate on VAD in 1997. What followed were decades of failed law reform attempts around assisted dying.

### A. The Rights of the Terminally Ill Act 1995 (NT)

The first VAD Bill in Australia was introduced into the Legislative Assembly of the Australian Capital Territory as early as 1993.^9^ Yet, legislating VAD was seen as ‘inopportune’ at the time, and the Bill did not become law.^10^

In 1995, a Private Member’s Bill on the *Rights of the Terminally Ill* was tabled in the Northern Territory.^11^ As pointed out above, the Northern Territory is a Territory on the Australian mainland and has self-government powers since the introduction of the *Northern Territory (Self Government) Act 1978* (NT).^12^ While the Northern Territory is a vast Federal Territory, it was scarcely populated in the 1990s with a population of just under 1% of the total population of Australia.^13^ Its lower house, the Legislative Assembly, consisted of only twenty-five members.

On 22 February 1995, Marshall Perron, Chief Minister of the Northern Territory at the time, introduced the *Rights of the Terminally Ill* Bill as a Private Member’s Bill.^14^ Private Members’ Bills are often introduced by backbenchers, but can also be introduced by Ministers, who act in their ‘individual capacity rather than as member representing the Government or Opposition’.^15^ While parliament’s agenda is generally dominated by the government, Members of Parliament can make independent policy proposals via Private Members’ Bills. Bowler notes that Private Members’ Bills ‘represent an area in which MPs have more freedom to pursue their own goals rather than those of the party or, more specifically, those of the government’.^16^ In May 1995, Perron resigned as Chief Minister as well as a Member for Fannie Bay ‘to minimise any influence he had over Members in the exercise of their choices’.^17^

Although the *Rights of the Terminally Ill* Bill was not a Government Bill and Perron resigned as Chief Minister and Member after its introduction, its success is frequently attributed to Perron’s lead role in the process,^18^ his ‘hard work and personal commitment’,^19^ and perhaps most importantly the fact that the Bill was ‘backed by his own power and influence’.^20^

During the presentation of the Bill to the Northern Territory Legislative Assembly, Perron explained that assisted dying was a ‘human rights issue’^21^ and concluded that:

[t]he focus of this legislation is to give those who suffer the right to choose a death with dignity, to bring to an end the torture many endure on their death-bed, and for that to be done legally without fear of prosecution for those doctors or nurses who may assist a patient in this desire.^22^

The matter was referred to a Parliamentary Select Committee on Euthanasia for inquiry and its report was tabled in the Legislative Assembly on 16 May 1995. The Committee did not comment on whether euthanasia should be legalised but made amendments to the proposed legislation and considered the improvement of palliative care.^23^ After 2 days of deliberations in the Legislative Assembly, the *Rights of the Terminally Ill Act 1995* (NT) was passed on 25 May 1995 by 15 to 10.^24^ The Northern Territory legislation was the first law in the world to legalise assisted dying and referred to by some as a ‘watershed moment in the history of euthanasia’.^25^ Withstanding two attempts to repeal or terminate the Act,^26^ it commenced operation on 1 July 1996. According to the Act, a patient over 18 years of age,^27^ who suffers from a terminal illness for which there is no cure,^28^ may request assistance in dying from a medical practitioner.^29^ Assistance is defined as including ‘the prescribing of a substance, the preparation of a substance and the giving of a substance to the patient for self administration, and the administration of a substance to the patient’.^30^ Apart from the original medical practitioner, another two medical opinions must be obtained supporting the request for assisted dying.^31^ The death must be subsequently reported to a Coroner by sending them a copy of the death certificate as well as all relevant medical records relating to the terminal illness and death of the patient.^32^ The Act removed criminal liability for those medical practitioners who acted ‘in good faith and without negligence in compliance with this Act’.^33^

Commentators note that, at the time, the Northern Territory legislation and the assisted dying it permitted attracted the full range of responses from ‘approval to vociferous condemnation’.^34^ For example, a 1995 Australian public opinion poll showed that 75% of participants supported ‘the introduction of a law which protects doctors who assist terminally ill patients who choose to end their own lives’.^35^ On the other hand, for instance, the Central Land Council, which represents the Indigenous population in the Northern Territory, strongly opposed the law deeming it ‘culturally inappropriate’.^36^

Between September 1996 and March 1997, four adult individuals with a terminal illness successfully accessed assisted dying in the Northern Territory.^37^ In 1996, the Northern Territory Supreme Court in *Wake and Gondarra v The Northern Territory of Australia*^38^ had to decide on the validity of the Act. The question to be addressed was, *inter alia*, whether the Northern Territory had competency to legislate on the matter of assisted dying. With a majority of two to one, the Supreme Court rejected the challenge to the legislation and held the law valid.^39^ While the judgment was subsequently appealed to the High Court of Australia, the application for special leave was stood over in November 1996 due to the below developments in the Federal Parliament.^40^

In September 1996, Senator Kevin Andrews, a ‘Catholic backbencher’,^41^ introduced the *Euthanasia Laws Bill* as a Private Member’s Bill into the lower house of the Australian Parliament, the House of Representatives. The Bill has since been termed ‘the Andrews’ Bill’.^42^ The Bill aimed to remove the Territories’ competency to legislate on matters concerning assisted dying. Andrews explained the legislation was necessary to protect vulnerable individuals in the Northern Territory who otherwise would be subject to abuse, coercion, and loss of autonomy.^43^

According to section 122 of the Australian Constitution, the Federal Government has the power to make laws for any Territories;^44^ a power it invoked in the case of assisted dying. Whether this was an appropriate use of this power, however, has been questioned in debates surrounding subsequent law reform attempts seeking to repeal the legislative limitations imposed on the Territories in this space.^45^

Interestingly, a public opinion poll at the time found that three-quarters of Australian voters opposed the passing of the Andrews’ Bill.^46^ Yet, an alliance of mostly Catholic politicians from both major political parties, medical practitioners and anti-assisted dying lobby groups, including *Euthanasia No!*, established solely for the purpose of preventing euthanasia in Australia,^47^ successfully campaigned for the passing of the Andrews’ Bill. The campaign initially relied on the ‘slippery slope’ argument discussed further below and ultimately strategically focused on ‘lethal injection’ and the sanctity of life.^48^

The Bill passed the House of Representatives in December 1996 and the Senate subsequently referred it for consideration to its Legal and Constitutional Affairs Legislation Committee. The Committee received more than 12,000 submissions, the large majority of which expressed opinions against assisted dying.^49^

In March 1997, less than 2 years after the enactment of the *Rights of the Terminally Ill Act*, and only 9 months after the law commenced operation, the Federal Government passed the *Euthanasia Laws Act 1997* (Cth), invalidating the Northern Territory law and depriving the Territories of the power to legislate on assisted dying in future.^50^ The Act, as Funk explains, was passed because of ‘public and political fears’^51^ in relation to assisted dying.

The Act led to the amendment of the *Northern Territory (Self Government) Act 1978* (NT) through the introduction of s 50A(1), which sets out that the Northern Territory Legislative Assembly does not have jurisdiction over laws

which permit or have the effect of permitting (whether subject to conditions or not) the form of intentional killing of another called euthanasia (which includes mercy killing) or the assisting of a person to terminate his or her life.

The *Rights of the Terminally Ill Act* has never been repealed by the Northern Territory Legislative Assembly but has continued to exist—on paper only—since the passing of the Federal law on 27 March 1997.^52^

After the passing of the *Euthanasia Laws Act 1997* (Cth), some commentators noted that ‘no legislation will end this debate [on assisted dying] until the fears embodied in the slippery slope argument can be dealt with in a manner that will allay the most pressing of these fears’.^53^ The slippery slope argument generally revolves around the assumption that decriminalising assisted dying ‘will lead down a psychological and philosophical *slippery slope*’^54^ ultimately broadening the scope of assisted dying to ‘those most in need of support and care’ risking ‘non-voluntary’ and ‘involuntary’ assisted deaths.^55^ The fears associated with allowing assisted dying appear not to have been alleviated in subsequent decades as no assisted dying legislation was successfully passed in Australia until 2017.

### B. Law reform attempts between 1993 and 2017

In 1997, Magnusson predicted that legalising VAD in Australia by introducing ‘a-right-to-die within a framework of safeguards’ was an ‘inevitable development’.^56^ Yet, this predicted development was halted for two decades.

The years following the introduction of the *Euthanasia Laws Act 1997* saw six unsuccessful law reform attempts aimed at removing the legislative limitations imposed on the Territories in the context of assisted dying.^57^ Overall, between 1993 and 2017, fifty-seven Bills dealing with VAD law reform were unsuccessfully introduced in Australian jurisdictions.^58^ Few managed to reach the Committee or third reading stage.^59^ The below ponders possible reasons for this development.

Magnusson explains that ‘[w]ithin a parliamentary context, the reform process ultimately relies upon (i) broad public support; (ii) successful advocacy by major players in the political debate; and (iii) parliamentary facilitators’.^60^

In relation to the first point, it is important to note that assisted dying in Australia had garnered broad community support since the early 1990s. While limited academic studies have examined the public attitudes towards VAD in Australia in past decades, public opinion polls show that the large majority of surveyed participants had been supportive of assisted dying for decades.^61^ For example, in public opinion polls across Australia throughout the 1990s more than 70% of participants responded ‘yes’ to the Morgan Poll^62^ question of whether a medical practitioner should, upon request, be allowed to give a lethal dose to a ‘hopelessly ill patient, experiencing unrelievable suffering’ without the possibility of recovery.^63^

Yet, regarding Magnusson’s second and third points, it appears that politicians were not in favour of such law reform. All failed Bills relating to aspects of VAD were Private Members’ Bills^64^ and thus introduced without government backing. While the first successful assisted dying law in the Northern Territory was also introduced as a Private Member’s Bill, the key difference between this law and subsequent unsuccessful Private Members’ Bills is as follows. The Northern Territory Bill was introduced by the Chief Minister, albeit in his individual capacity rather than as a representative of the government. Yet, the Bill, although not a Government Bill, was nevertheless backed by his political influence and the power stemming from his role.

Even though the major political parties had no policies on VAD in the past and conscience votes were permitted each time,^65^ Willmott and White explain that there was a correlation between ‘party affiliations and voting preferences’ with conservative party members generally voting against assisted dying law reform.^66^ Similarly, Ho and Penney concluded in 1992 that the degree of conservatism generally predicted attitudes towards assisted dying.^67^ In their research published in 2018, McGee and others examined the attitudes of Australian politicians towards assisted dying since the introduction of the first euthanasia Bill.^68^ The study highlights that ‘the votes of individual members tend to strongly correlate with their party affiliations and coalitions’ and that Liberal Party members were more likely to vote against assisted dying while Labor Party members were more likely to be in support.^69^ Their research shows that in 1996 arguments against legalising assisted dying advanced by politicians in parliaments across Australia significantly outweighed those in its support.^70^ By December 2017, however, this was no longer the case as the frequency with which politicians throughout Australia argued for and against assisted dying was more closely aligned.^71^ This led to the following status quo of VAD laws in Australian States.

## III. THE CURRENT STATUS QUO OF VAD LAWS IN AUSTRALIA

Traditionally, each Australian State and Territory enacts their own criminal offences. In addition, Federal criminal law exists. Some Australian States base their criminal law on criminal codes,^72^ while others rely more heavily on common law.^73^ In relation to VAD, the following criminal law situation exists across Australian jurisdictions.

### A. General liability under criminal law

In all Australian jurisdictions, taking one’s own life or attempting to do so no longer gives rise to criminal responsibility.^74^ However, aiding, counselling, or inciting another person to kill himself or herself is generally a criminal offence.^75^ As a general rule, individuals cannot lawfully assist others in ending their lives.^76^

Moreover, intentionally ending the life of another, even upon the person’s request and with their consent, generally amounts to murder, punishable with a maximum or mandatory penalty of life imprisonment depending on the jurisdiction.^77^ Where the required intent for murder cannot be established or certain partial defences apply, criminal responsibility remains for manslaughter, punishable with a maximum sentence of life imprisonment.^78^

To prevent or limit criminal responsibility for participation in VAD, all Australian States have now decriminalised relevant conduct by enacting specific VAD frameworks. Conduct which does not comply with the respective legislation remains subject to criminal liability.

### B. VAD laws in all six Australian states

VAD laws were first introduced in Victoria^79^ in 2017 and have been called ‘ground-breaking legislation’^80^ and ‘an historic change of ethical and political significance’.^81^ VAD laws were subsequently introduced in 2019 in Western Australia,^82^ in April 2021 in Tasmania,^83^ in July 2021 in South Australia,^84^ in September 2021 in Queensland,^85^ and in May 2022 in New South Wales.^86^

A person seeking access to VAD in participating jurisdictions must meet specific requirements including being diagnosed with a disease, illness or medical condition that is advanced, progressive, and expected to cause death^87^ within 6 months unless suffering from a neurodegenerative disease, in which case it is no more than 12 months until expected death in Victoria, Western Australia, Tasmania, South Australia, and New South Wales.^88^ In Queensland, death must be expected to occur within 12 months regardless of the nature of the illness, disease, or condition.^89^ In addition, according to the relevant legislation, the illness must cause suffering which the person considers to be intolerable.^90^ The person seeking access to VAD must also: be an Australian citizen or permanent resident and have been ordinarily resident of the respective State for at least 12 months at the time of first requesting VAD,^91^ be 18 years or older,^92^ act voluntarily, with decision-making capacity in relation to VAD, and their request must be enduring.^93^

## IV. FACTORS CONTRIBUTING TO RAPID LAW REFORM

The question arises why, after two decades of failed law reform attempts, all six Australian States have adopted laws allowing VAD in only 5 years since 2017. This is explored in light of Magnusson’s three points underpinning successful parliamentary reform processes, namely ‘(i) broad public support; (ii) successful advocacy by major players in the political debate; and (iii) parliamentary facilitators’^94^ in the context of the first Australian jurisdictions introducing VAD laws. In the context of subsequent States, a fourth point is explored which concerns the potential impact of the precedent set by successful law reform in the first Australian jurisdictions.

### A. Broad public support

In relation to the first point, community support for VAD in Australia continued to grow since the 1990s, where it was already more than 70% in favour of assisted dying. The national support of assisted dying was 73% in the 2002 Morgan Poll. In 2007, a Newspoll survey showed that 80% of Australians were in favour of assisted dying for terminally ill persons, which increased to 85% in the 2009 Newspoll.^95^ In 2016, 75% were in favour of letting terminally ill patients legally end their own lives with medical assistance.^96^ As public opinion was already strongly in favour of VAD in the 1990s, this factor alone cannot sufficiently explain the rapid law reform success in recent years.

Magnusson’s second and third points relate to the involvement of parliamentary facilitators and the advocacy of major players in the political sphere, which will be discussed below.

### B. Parliamentary facilitators

#### 1. Government support in Victoria and Western Australia

For the first time in Australian history, in 2017 a VAD Bill was backed by the government. The Vic VAD Bill was introduced as a Government Bill and not as a Private Member’s Bill.^97^ Due to the government backing, the Vic VAD Bill attracted higher levels of publicity and was perceived with more credibility than prior Private Members’ Bills which remained unsuccessful.^98^ O’Connor and others agree that ‘[t]he Victorian government’s leadership and cross-party support for the development of this legislation contrasts with the many unsuccessful attempts by individual politicians in Australia to put private member’s bills into legislation’. They conclude that government support seems to have been essential in the development of a high-quality Victorian Bill.^99^

It is unclear why a VAD Bill found government support in 2017. Prior to the Victorian state election in 2014, the government did not foreshadow the introduction of VAD laws.^100^ It may have been to do with the fact that the Victorian government at the time was a Labor government with an emphasis on social reform. This included, for example, enhancing access to abortion services. Inquiring into VAD was in line with the government’s general social agenda.^101^ As Duckett explains, the Premier, the Minister for Health and the Special Minister of the State strongly supported the Bill and ‘played important roles in determining the government’s response and in negotiating the bill’s passage’.^102^ The Bill also found support from the Leader of the Opposition in the Upper House.^103^ Others opine that the Victorian Government ultimately backed the Bill as the Greens had announced they would put forward a VAD bill should the government fail to do so. This pressure from inside parliament, so the argument goes, may have *inter alia* ‘pushed the government to act, because regardless of the government’s actions there was going to be a debate on dying with dignity laws in this term of Parliament’.^104^

The significance of the Bill’s government support was acknowledged by members during parliamentary debates. For example, during the Bill’s second reading in the Legislative Council, it was pointed out that the legislative process had been ‘driven by the government’ while being ‘supported by the minor parties’.^105^ One member noted that it was better that the government had used the ‘far greater resources at their disposal’ and taken ‘ownership of this legislation’.^106^

The Parliament of Western Australia, the next State to introduce VAD laws, established a Joint Select Committee on End-of-Life Choices on 23 August 2017 and requested that it report by 23 August 2018. In its 2018 report ‘My Life, My Choice’, the Committee pondered, *inter alia*, why only two VAD law reforms, namely in Victoria and the Northern Territory, had been successful in Australia until that time. It concluded that ‘[i]t is clear that government support is necessary if reforms to end of life laws are to be achieved’.^107^ The Committee therefore considered government support essential in the successful passing of VAD laws in Western Australia. The WA VAD Bill was subsequently introduced as a Government Bill.

#### 2. Consultation and inquiry into VAD in Victoria and Western Australia

Duckett submits that ‘extensive public consultation and the ability to air the tragic stories associated with failures of end-of-life care’ may have been an important factor in swaying lawmakers to pass the legislation.^108^ Moreover, White and others highlight the importance of ‘good process’ in achieving law reform including extended consideration periods, assessment of evidence of existing practices including the potential need for reform, open dialogue with the public and profession and clear communication.^109^

The reform process in Victoria was well-funded and allowed for extensive research ahead of the preparation of the legislation, ultimately resulting in the production of high-quality materials relating to assisted dying.^110^ The wide consultation and extensive Victorian reform process may be a key reason why VAD law reform was successful in Victoria.^111^ To illustrate this point further, prior to the introduction of the first Australian VAD Act in Victoria in 2017, the Victorian Legal and Social Issues Committee of the Legislative Council was tasked with an ‘Inquiry into End of Life Choices’ in 2015 focusing on ‘the need for laws in Victoria to allow citizens to make informed decisions regarding their own end of life choices’.^112^ The review considered existing legislative frameworks relating to end-of-life decision making in Australia and internationally while also seeking community views on the necessity of allowing broader access to end-of-life choices. The Committee received 1,037 written submissions in response to its inquiry with the majority of individual submissions focusing on VAD.^113^ This was followed by 17 days of formal hearings across Victoria and, in early 2016, with Committee member visits to international jurisdictions permitting VAD in different forms to gain an in-depth understanding of the operation of these schemes.^114^ The Committee issued a Final Report in 2016 in support of the introduction of VAD laws in Victoria and made recommendations towards a legislative framework.^115^

Due to the Committee’s inquiry and recommendations, the Victorian government agreed to support the development of a Bill. Some Members of Parliament at the time considered the work of the Legal and Social Issues Committee ‘integral to progressing this issue’ concluding that ‘without the committee’s work and the recommendation in support of voluntary assisted dying, this bill would not be before this Parliament’.^116^ The government established a Ministerial Advisory Panel on Voluntary Assisted Dying in 2016, tasked with commenting on avenues for the safe implementation of VAD in Victoria, including legal and policy issues. The consultation process included expert consultation via the release of a discussion paper.^117^ In its 2017 final report, the Panel recommended a VAD framework and outlined access criteria and processes.^118^ After this recommendation, the Labor government introduced the Vic VAD Bill into parliament, which was subsequently passed in November 2017.

A similarly extensive consultation and inquiry process into VAD occurred in Western Australia. In August 2017, the Western Australian Parliament appointed a joint select committee tasked with inquiring into end-of-life choices. During their year-long inquiry, the Committee received over 700 submissions, held 81 hearings, and heard from 130 witnesses.^119^ In August 2018, the Committee handed down its final report to both Houses of Parliament recommending the development and introduction of VAD legislation.^120^ In response, the Western Australian government undertook to introduce a VAD bill and appointed a Ministerial Expert Panel to assist with the development of said legislation.^121^ The Panel also consulted extensively receiving 541 submissions and hearing from 867 participants and organisations during the consultation process.^122^

### C. VAD advocacy

In relation to Magnusson’s point of successful advocacy by major players the below considers the role of VAD advocacy groups including their use of media and how this may have impacted the political debate on VAD in Victoria and Western Australia.

Duckett points out that opposition to VAD had always been ‘well-organised and vocal’ and that in the past this ‘concentrated opposition’ was able to overwhelm voices supporting assisted dying in Australia.^123^ Yet, by the mid-2010s high-profile assisted dying support organisations were heavily campaigning in favour of VAD laws, thus creating a concentrated support effort in the passing of the legislation in Victoria and subsequently Western Australia.

Dying with Dignity Victoria,^124^ originally founded in 1974 with the aim to bring about law reform in the end-of-life arena, increased its campaigning efforts in Victoria to influence parliamentary debate on VAD between 2015 and 2017 by, *inter alia*, providing extensive written submissions and invited oral submissions in assisted dying inquiries.^125^

Around the same time as Dying with Dignity increased their advocacy efforts in Victoria, Go Gentle Australia was launched in 2016. The organisation was established by Andrew Denton with the aim to promote choice at the end of life, including the option of voluntary assisted dying.^126^ Denton is a well-known Australian television presenter and comedian and was the host of a weekly television program on the Australian ABC. Go Gentle Australia was founded on the back of Denton’s popular podcast series ‘Better Off Dead’, launched in 2015, which featured accounts of assisted dying from around the globe.^127^ Denton explains that he was inspired to launch the podcast after seeing his father die a painful death.^128^

Go Gentle Australia made extensive submissions to government inquiries on assisted dying in Victoria.^129^ Moreover, Denton published comments and opinion pieces in support of VAD in major Australian newspapers in the 2016–2017 period including *The Australian*^130^ and *The Sydney Morning Herald*.^131^ Newspaper reporting also covered his VAD advocacy efforts in the Victorian context.^132^ Go Gentle commissioned several polls in 2017 surveying Victorian adults on their views on assisted dying. All surveys found overwhelming support for introducing the right to assisted dying for terminally ill adults in Victoria.^133^

The groups’ advocacy efforts did not go unnoticed during the 2017 Victorian VAD law reform process as illustrated by parliamentary debates on the issue. For example, during the second reading of the Vic VAD Bill in the Legislative Council, one Member noted that they wanted to ‘place on record the very thoughtful advocacy of Andrew Denton, Paul and the team at Go Gentle, who have been working very hard in terms of, obviously, the case for this legislation’^134^ with another remarking that they wanted to ‘acknowledge the tireless work of Dying with Dignity Victoria, Andrew Denton and Go Gentle Australia, who have campaigned so strongly and respectfully for this legislation’.^135^

Particularly the results of the Go Gentle surveys of Victorian voter preferences on VAD seem to have influenced political debate. One Member of the Legislative Council commented that they found it especially interesting where the poll revealed that ‘84 per cent of our constituents who vote support voluntary assisted dying’^136^ while a Member of the Legislative Assembly noted that it had been difficult to identify the level of support in their electorate but that Go Gentle Australia had commissioned polling in their particular electorate identifying ‘an almost unbelievably high level of support for the bill’.^137^

Members concluded that pressure from inside parliament but also ‘pressure from advocates in the community such as Dying with Dignity Victoria and Go Gentle Australia, and the extensive committee report pushed the government to act …’.^138^

Go Gentle Australia’s advocacy efforts were again influential in the Western Australian law reform context with Members of the Legislative Council noting that they wanted to acknowledge the ‘amazing amount of work’ done by Go Gentle Australia and the important materials provided to politicians.^139^ As in Victoria, Go Gentle Australia conducted polls on whether Western Australians were in favour of introducing VAD. During parliamentary debates, Members explicitly referred to these polls and emphasised the overwhelming community support for VAD these polls identified.^140^ One concluded that members of Go Gentle Australia had

been a critical part in mobilising public passion for this change and providing counsel to members of Parliament on how the fight would play out. They helped us understand the challenges and techniques that opponents used in every jurisdiction in which such legislation has been introduced. Certainly, being forewarned definitely helped us to be forearmed.^141^

The above suggests that parliamentary facilitators and VAD advocacy by key players were important factors in the VAD reform process in Victoria and Western Australia.

### D. Successful law reform as precedent

One factor contributing to successful law reform in subsequent Australian States may have been the enactment of VAD laws in the previous States and the precedent this created. It is noteworthy that subsequent reform processes in some States were much speedier than in the first two jurisdictions. This may be to do with the fact that subsequent States were able to draw on the work already carried out by committees in this space and the experiences these States had made with the operation of VAD laws in practice.^142^

In Tasmania, the VAD Bill was introduced as a Private Member’s Bill on 27 August 2020 and law reform had occurred by April 2021. While the Tasmanian Policy Exchange at the University of Tasmania undertook an *Independent Review of the End of Life Choices (Voluntary Assisted Dying) Bill*, no parliamentary committee was tasked with a review of VAD.^143^ At the time of the publication of the independent review’s report in February 2021, the Bill had already passed the Lower House and was ultimately passed in April 2021. During parliamentary debates on VAD in Tasmania, some Members particularly pointed to the legislative situation in Victoria and Western Australia to support the argument that assisted dying laws should be introduced in Tasmania.^144^ One opined that ‘VAD has been operating in Victoria for a year and there have been no significant problems. The Victorians are currently helping the Western Australians to gear up and this help is valuable and appreciated. If the bill is passed in Tasmania we can anticipate similar help’.^145^ Another remarked that ‘[b]oth the Victorian and WA parliamentary inquiries found that there is no evidence for the arguments of opponents of voluntary assisted dying such as coercion, the slippery slope or elder abuse’.^146^

In South Australia, the VAD law reform process was also much shorter than in Victoria and Western Australia. The VAD Bill was introduced to both Chambers of the South Australian Parliament on 2 December 2020 as a Private Member’s Bill and received Assent by the Governor of South Australia on 24 August 2021. No extensive inquiry and consultation process into VAD laws comparable to that in Victoria or Western Australia occurred. Again, during parliamentary debates, some looked towards the already introduced VAD laws in other Australian jurisdictions with the Premier at the time commenting that ‘the deliberations and decisions in other jurisdiction’ have developed ‘a well-considered Australian model and nationally consistent legislation in this very sensitive area of health law’. Therefore ‘South Australia can have confidence in joining other states in enacting a model law for voluntary assisted dying. Such a model law includes strict eligibility and approval conditions’.^147^

While Queensland did not enact VAD laws until 2021, the Queensland Legislative Assembly had already tasked the Queensland Parliament Health, Communities, Disability Services and Domestic and Family Violence Prevention Committee in 2018, with ‘seeking Queensland’s view on the desirability of a voluntary assisted dying scheme’. The Committee’s extensive report including recommendations was published in March 2020.^148^ The Queensland Law Reform Commission was subsequently called upon to develop and recommend draft VAD legislation for Queensland. Its final report was published in 2021,^149^ which led to the introduction of the Qld VAD Act based on a Government Bill later that same year. During parliamentary debates, Members referenced the situation in other Australian jurisdictions to justify the introduction of VAD laws in Queensland. During the second reading of the VAD Bill in the Legislative Assembly, for example, the Deputy Premier noted that ‘Queensland is one of only two remaining states in Australia to have not yet enacted voluntary assisted dying legislation. The time has come to recognise that Queenslanders who are suffering and dying deserve to have choice and autonomy about their end of life’.^150^

The last Australian State to decriminalise VAD was New South Wales. Similar to South Australia and Tasmania, the Bill was introduced as a Private Member’s Bill on 14 October 2021 and had passed both Houses by May 2022. While the Bill was debated for 8 days in the Legislative Assembly leading to a suite of changes, no inquiry into VAD comparable to Victoria and Western Australia occurred in New South Wales.^151^ During the second reading speech, the Hon Adam Searle emphasised that the situation in New South Wales was different now as ‘today every other State has already enacted voluntary assisted dying laws. In Western Australia and Victoria those laws are safely in operation today’.^152^ He pointed out that looking at the situation in Victoria provided evidence that no ‘terrible misapplications or exploitations of the law’ would likely occur in New South Wales as there had not been ‘cases of pressure or duress, no access by ineligible people and no misuse of the lethal substances’ in Victoria.^153^

Tracing the history and development of VAD laws in Australia shows that government backing, extensive review and consultation processes and concentrated lobbying efforts by high-profile advocacy groups appear to have played an important role in the successful passing of VAD laws in the first States—Victoria and Western Australia. It was also important in Queensland, where the inquiry and consultation process had already commenced before VAD law reform occurred in Victoria. Some of these factors became less important in the successful passing of VAD laws as time went on. For example, in Tasmania, South Australia, and New South Wales, the VAD Bills were introduced without government backing as Private Members’ Bills and no extensive parliamentary inquiries into assisted dying and consultations comparable to the ones in the first jurisdictions took place. It should be noted that during parliamentary debates in all subsequent jurisdictions contemplating law reform, explicit reference was made to the situation in those Australian jurisdictions, which had already introduced VAD laws. This suggests that already existing laws in Australian jurisdictions instilled confidence in lawmakers to bring about VAD law reform in their own jurisdiction.

What impact these developments may have on VAD law reform in the Australian Territories is discussed below.

## V. FUTURE VAD LAW REFORM IN THE AUSTRALIAN TERRITORIES

As outlined above, with the introduction of the *Euthanasia Laws Act 1997* (Cth), the territories were deprived of the power to legislate on VAD. Subsequent years saw six unsuccessful attempts at removing the legislative limitations imposed on the Territories.^154^ Making VAD lawful in the territories is a two-fold process first requiring the overturning of the above Commonwealth legislation and secondly introducing Territory VAD laws.

### A. Overturning the Euthanasia Laws Act 1997 (Cth)

In relation to the first step to VAD law reform in the Territories, that is overturning the *Euthanasia Laws Act 1997* (Cth) limiting the Territories’ legislative powers regarding VAD, the *Restoring Territory Rights Bill 2022* was introduced into Federal Parliament and passed the lower house in August 2022. The Bill proposed to remove the restrictions contained in the *Australian Capital Territory (Self-Government) Act 1988* and the *Northern Territory (Self-Government) Act 1978* currently preventing the Territories from passing legislation on VAD. The debate on Territory rights began in the Senate in September 2022 and the law was passed on 1 December 2022.^155^ Territories are therefore able to legislate on assisted dying for the first time in over two decades.

### B. VAD laws in the territories

Secondly, to make VAD lawful in the Territories, VAD laws would have to be passed in each jurisdiction. At the time of writing, debate on VAD law reform has commenced in the ACT while the Northern Territory has not yet stated any plans for future legislation.^156^ The question arises whether VAD law reform attempts in the Territories would likely be successful; especially considering the large number of failed past law reforms in this space.

In 1996, in the context of arguments against assisted dying Somerville noted that:

[i]t is not possible to argue against euthanasia from an empirical base. Euthanasia constitutes a very serious criminal offence in the vast majority of jurisdictions and, consequently, research cannot be undertaken to produce ‘hard’ evidence of the impact that legalising it would have.^157^

She emphasised that this ‘leaves opponents of legalisation open to criticism that their arguments are purely speculative and lacking in scientific rigour’.^158^

The very same argument could have been made in support of assisted dying at the time. No empirical evidence concerning the safe operation of assisted dying laws was available as the conduct was generally criminalised. This point is explained further by Kerridge and Mitchell, who, during the time of the brief operation of the *Rights of Terminally Ill Act 1995* (NT), pointed out that there was no existing knowledge yet as to ‘whether support for voluntary euthanasia is based upon a dangerously naive view of rights, autonomy and society, or whether objections to legalising voluntary euthanasia are based on illusory, rather than real, slippery slopes’.^159^

Since then, however, the situation has changed significantly in Australia, where VAD legislation is expected to be in operation in all States by late 2023. White and others note that lawmakers’ ability to draw on existing evidence supporting the premise that VAD legislation can safely operate in practice discredits the well-rehearsed argument that VAD laws create risks for vulnerable individuals.^160^ The researchers argue in an international context that this so-called ‘shrinking battlefield’, namely the reduction of arguments against VAD due to emerging empirical evidence concerning its safe and efficient practical operation, ‘has shaped the nature of assisted dying debates and made reform more likely’.^161^

With VAD law reform becoming increasingly reliant on evidence from those jurisdictions where VAD is already lawful,^162^ Territory lawmakers are in the unique position to draw on empirical evidence from Australian States. The currently available reports from bodies overseeing the operation of VAD in Victoria and Western Australia, for example, do not suggest that the legislation operates unsafely.

In Victoria, where VAD legislation has been in operation since 2019 and thus the longest in Australia, the Voluntary Assisted Dying Review Board reports on the operation of the Vic VAD Act at intervals, initially biannually and now annually. In its most recent report published in September 2022, the Board reflected on the past 3 years of operation and concluded that ‘[t]he most significant matter to report is that voluntary assisted dying in Victoria continues to operate safely and lawfully’.^163^ Between 1 July 2021 and 30 June 2022, a total of 401 deaths of applicants with assisted dying permits were recorded.^164^ During the same time frame, the Board identified only four cases which it deemed non-compliant with the Vic VAD Act. Three cases were deemed non-compliant due to delays in returning any outstanding lethal substance to the pharmacy by the contact person, which must occur within 15 days of the death of an applicant.^165^ The fourth case was a violation of the Vic VAD Act section prohibiting someone who signs on behalf of an applicant to also be a witness to the document. The Board’s investigation determined this to be an oversight and the case to be clinically appropriate. Consequently, no action was taken.^166^

The WA VAD Act came into operation on 1 July 2021 and the Western Australian Voluntary Assisted Dying Board published its first annual report on the operation in November 2022.^167^ During the time frame,190 deaths were recorded following the administration of a voluntary assisted dying substance.^168^ The WA Board made only one referral to the Chief Executive Officer of the Department of Health relating to the timeliness of an authorised disposal of a voluntary assisted dying substance.^169^ It concluded overall that voluntary assisted dying had become ‘accessible and safe for Western Australians’.^170^

As the VAD Act in Tasmania only commenced in 2022, the Voluntary Assisted Dying Commission Tasmania has not yet published its first annual report on the operation of VAD legislation. Yet, a March 2023 update from the Commission concluded that the ‘Act is continuing to operate as intended and the safeguards applied through the legislation are working’.^171^

The available empirical evidence on the safe operation of the Vic and WA VAD Acts for several years may make a compelling argument in legislative debates on assisted dying and likely sway lawmakers to pass relevant legislation in the Territories shortly. Similarly, White and Wilmott predicted in 2018 that after the Victorian VAD law reform, other States and Territories would follow^172^ because VAD in Australia was ‘a train that has left the station’.^173^

## VI. CONCLUSION

After the short-lived operation of the Northern Territory’s *Rights of the Terminally Ill Act 1995*, Australia faced more than two decades of failed law reform attempts on VAD. This article highlighted that while public support for VAD law reform has been strong in Australia since the 1990s, politicians, especially those with more conservative attitudes, did not appear in favour of decriminalising assisted dying. It was not until 2017 that the first Australian VAD law reform backed by a government occurred in Victoria. What followed was a chain of rapid VAD law reforms in all Australian States in only 5 years. In comparison to past failed reform attempts, the Victorian reform process was characterised by government support, a well-funded, extensive and lengthy consultation process and concentrated advocacy efforts from high-profile organisations in support of VAD. Government support, lengthy consultation, and concentrated advocacy efforts can also be detected in the WA reform processes. Government support, extensive consultation, and inquiries into VAD became less important in the law reform context as time went on. All successive VAD Bills but for the Qld VAD Bill were introduced as Private Members’ Bills without government support, and no parliamentary review or inquiry into VAD comparable to that in Victoria and Western Australia took place. Rather, subsequent States pointed to VAD legislation already enacted or in operation in other Australian States. This suggests that VAD law reform garnered momentum after the first two States had passed the respective legislation ultimately creating a precedent for law reform in subsequent States.

The *Restoring Territory Rights Bill 2022* has paved the way for VAD law reform in the Territories. The enactment of VAD laws in all Australian States and its commencement in some has generated the first empirical evidence supporting its safe and efficient operation. On this basis, it is easy to imagine that successful VAD law reform will soon follow in the Australian Capital Territory and the Northern Territory ultimately making VAD lawful in all Australian jurisdictions.

## Footnotes

1 New South Wales (NSW), Queensland (Qld), South Australia (SA), Tasmania (Tas), Victoria (Vic), and Western Australia (WA).

2 Located on the Australian mainland: The Northern Territory and the Australian Capital Territory.

3 In 1995, the Northern Territory enacted the *Rights of the Terminally Ill Act 1995* (NT) making assisted dying lawful as the first jurisdiction in the world. However, in 1997 less than 2 years after its enactment, the Federal Government passed the *Euthanasia Laws Act 1997* (Cth), depriving the Territories of their power to legislate on VAD. This also invalidated the Northern Territory legislation. This development is discussed in detail below.

4 In December 2022, the *Restoring Territory Rights Bill 2022* (Cth) which repealed the limitations on the two Territories was passed; Josh Burler, ‘Territory Governments Regain Right to Make Assisted Dying Laws After Senate Vote’ *The Guardian* (1 December 2022) <https://www.theguardian.com/society/2022/dec/01/territory-governments-regain-right-to-make-assisted-dying-laws-after-senate-vote> (last accessed 6 September 2023); Lisa Visentin, ‘Push to Overturn 25-Year Ban on ACT, NT Legalising Assisted Dying Clears First Hurdle’ *The Sydney Morning Herald* (3 August 2022) <https://www.smh.com.au/politics/federal/push-to-overturn-25-year-ban-on-act-nt-legalising-assisted-dying-clears-first-hurdle-20220803-p5b6st.html> (last accessed 6 September 2023).

5
*Voluntary Assisted Dying Act 2017* (Vic) (‘Vic VAD Act’) s 3; *Voluntary Assisted Dying Act 2019* (WA) (‘WA VAD Act’) s 5; *Voluntary Assisted Dying Act 2021* (SA) (‘SA VAD Act’) s 3; *Voluntary Assisted Dying Act 2021* (Qld) (‘Qld VAD Act’) Sch1; *Voluntary Assisted Dying Act 2022* (NSW) (NSW VAD Act) Sch1. The Tasmanian Act defines VAD in s 5 *End-of-Life Choices (Voluntary Assisted Dying) Act 2021* (Tas) (‘Tas VAD Act’) as ‘the administration to a person, or the self-administration by a person, of a VAD substance under this Act’.

6 See Vic VAD Act ss 46, 48; WA VAD Act ss 56(1)(b), 59; Tas VAD Act s 86(3); SA VAD Act s 64; Qld VAD Act s 53; NSW VAD Act s 60. According to the Acts, the primary method is self-administration and practitioner administration is only possible where the person is incapable of self-administration (Victoria, South Australia) or where self-administration is inappropriate (Western Australia, Tasmania, Queensland). The NSW VAD Act provides a true choice between practitioner and self-administration. See Vic VAD Act s 48(3)(a); WA VAD Act s 56(2); Tas VAD Act s 86(5); SA VAD Act s 64(c)(i); Qld VAD Act s 50(2); NSW VAD Act s 57.

7 See Vic VAD Act ss 45, 47; WA VAD Act ss 56(1)(a), 58; Tas VAD Act s 91; SA VAD Act s 63; Qld VAD Act s 52; NSW VAD Act s 59.

8 Vic VAD Act ss 79–82; WA VAD Act ss 113–115; Tas VAD Act ss 133–139; SA VAD Act ss 94–97; Qld VAD Act ss 147–149; NSW VAD Act ss 130–133.

9
*Voluntary and Natural Death Bill 1993* (ACT).

10 Select Committee on Euthanasia Parliament of the Australian Capital Territory, Report: Voluntary and Natural Death Bill 1993 (1994) 6–7 <https://www.parliament.act.gov.au/__data/assets/pdf_file/0011/382196/Select_com_on_enthunasia.pdf> (last accessed 6 September 2023).

11 Northern Territory, Parliamentary Debates, Legislative Assembly, 22 February 1995 (Marshall Perron) <https://parliament.nt.gov.au/__data/assets/pdf_file/0009/367947/serial67speech.pdf> (last accessed 6 September 2023).

12
*Northern Territory (Self Government) Act 1978* (NT) s 6: ‘Subject to this Act, the Legislative Assembly has power, with the assent of the Administrator or the Governor-General, as provided by this Act, to make laws for the peace, order and good government of the Territory’; see also Robyn Smith, ‘Northern Territory Parliamentary Report’ (2008) 23(2) APR 261, 261.

13 In December 1995, the estimated resident population of the NT was just under 177.000. See <https://www.ausstats.abs.gov.au/ausstats/free.nsf/0/3B16263CBA8554E0CA25722500049503/$File/33117_1995.pdf> (last accessed 6 September 2023). See also John Fleming, ‘Death, Dying, and Euthanasia: Australia Versus the Northern Territory’ (1999) 15(3) Issues Law Med 291, 298.

14 Chief Minister is the Head of government of the Northern Territory.

15 Nicolee Dixon, ‘The Role of Private Members’ Bills’ (2004) 19(1) APR 88, 88.

16 Shaun Bowler, ‘Private Members’ Bills in the UK Parliament: Is There an “Electoral Connection”?’ (2010) 16(4) J Legis Stud 476, 476.

17 Jodie Kline, ‘The Andrews’ Bill: Compromised Citizenship or the Protection of Human Rights?’ (2002) 13 J North Territ Hist 49, 50.

18 Simon Chesterman, ‘Last Rights: Euthanasia, The Sanctity of Life, and the Law in the Netherlands and the Northern Territory of Australia’ (1998) 47 ICLQ 362, 384 n 132.

19 Roger Magnusson, ‘The Future of the Euthanasia Debate in Australia’ (1996) 20(4) Melb Law Rev 1108, 1126.

20 ibid.

21 Northern Territory, Parliamentary Debates, Legislative Assembly, 22 February 1995 (Marshall Perron) 1 <https://parliament.nt.gov.au/__data/assets/pdf_file/0009/367947/serial67speech.pdf> (last accessed 6 September 2023).

22 ibid 3.

23 Northern Territory Select Committee on Euthanasia, The Right of the Individual or the Common Good (May 1995) <https://parliament.nt.gov.au/__data/assets/pdf_file/0007/367963/vol1.pdf> (last accessed 6 September 2023).

24 Northern Territory, Extracts from the Parliamentary Record of the Debates of the Legislative Assembly on the *Rights of the Terminally Ill Bill 1995* on 24 and 25 May 1995 A.M., Legislative Assembly, 24–25 May 1995 <https://parliament.nt.gov.au/__data/assets/pdf_file/0003/367950/extracts.pdf> (last accessed 6 September 2023).

25 Garima Jain and Sanjeev Sahni, ‘Euthanasia: A Review on Worldwide Legal Status and Public Opinion’ (2018) 1 Criminol Crim Law Rev 63, 73.

26 Patrick Quirk, ‘Euthanasia in the Commonwealth of Australia’ (1998) 13(4) Issues Law Med 425, 429.

27 Terminally Ill Persons Act 1995 (NT) s 7(a).

28 Terminally Ill Persons Act 1995 (NT) s 7(b)(i)–(ii).

29 Terminally Ill Persons Act 1995 (NT) s 4.

30 Terminally Ill Persons Act 1995 (NT) s 3.

31 Terminally Ill Persons Act 1995 (NT) s 7(c).

32 Terminally Ill Persons Act 1995 (NT) s 14.

33 Terminally Ill Persons Act 1995 (NT) s 20.

34 Sharon Fraser and James Walters, ‘Death-Whose Decision? Physician-assisted Dying and the Terminally Ill’ (2002) 176 West J Med 120, 120.

35 Margo Kingston, ‘MP Rebels as Poll Reveals 75% of Voters Want Euthanasia Laws’ *The Sydney Morning Herald* (9 October 1996).

36 Stephen M Cordner, ‘Reactions to Australian State’s Euthanasia Law’ (1995) 345 Lancet 1561, 1561.

37 Fraser and Walters (n 34) 120, 121. For case studies on the respective deaths see David Kissane, Annette Street and Philipp Nitschke, ‘Seven Deaths in Darwin: Case Studies Under the Rights of the Terminally Ill Act, Northern Territory, Australia’ (1998) 352 Lancet 1097.

38 Unreported judgment, Supreme Court of the Northern Territory (Martin CJ, Angel and Mildren JJ), No 112 of 1996, 24 July 1996.

39 Martin CJ and Mildren J, Angel J dissenting.

40
*Wake v Northern Territory* High Court of Australia, No D10 (1996).

41 Roger Magnusson, ‘The Sanctity of Life and the Right to Die: Social and Jurisprudential Aspects of the Euthanasia Debate in Australia and the United States’ (1997) 6(1) Wash Int Law J 1, 61.

42 Kline (n 17) 49.

43 ibid 51.

44 Smith (n 12) 267. That power does not extend to States but is limited to Territories.

45 ibid.

46 Kingston (n 35).

47 Lyn Carson and Brette Blakely, ‘What Can Oregon Teach Australia About Dying?’ (2013) 6(2) J Polit Law 30, 40.

48 Chesterman (n 18) 390.

49 Stephen Duckett, ‘The Long and Winding Road to Assisted Dying in Australia’ (2019) 54 Aust J Soc Issues 386, 387.

50 Act No 17 of 1997. The Senate voted 38-33 in favour of the Andrews’ Bill.

51 Margaret Funk, ‘A Tale of Two Statutes: Development of Euthanasia Legislation in Australia’s Northern Territory and the State of Oregon’ (2000) 14(1) Temple Int Comp Law J 149, 178.

52 Smith (n 12) 261.

53 Funk (n 51) 179.

54 Ian Kerridge and Kenneth Mitchell, ‘The Legalisation of Active Voluntary Euthanasia in Australia: Will the Slippery Slope Prove Fatal?’ (1996) 22 J Med Ethics 273, 277.

55 ibid.

56 Magnusson (n 41) 80.

57 See Lindy Willmott and others, ‘(Failed) Voluntary Euthanasia Law Reform in Australia: Two Decades of Trends, Models and Politics’ (2016) 39(1) UNSWLJ 1, 9-10.

58 Andrew McGee and others, ‘Informing the Euthanasia Debate: Perceptions of Australian Politicians’ 2018 41(4) UNSWLJ 1368, 1374. Overall, between 1993 and 2017, 58 Bills had been introduced and the *Voluntary Assisted Dying Bill 2017* (Vic) passed in Victoria in late 2017. Between 1993 and 2015, 51 Bills had been unsuccessfully introduced see Willmott and others (n 57) 1, 10. Out of the 51 bills, 39 explicitly aimed at permitting VAD, see Ben White and Lindy Willmott, ‘Future of Assisted Dying Reform in Australia’ 42(6) 2018 Aust Health Rev 616, 617. Queensland was the only jurisdiction in which VAD law reform had not been attempted during this time frame.

59 Lindy Willmott and Ben White, ‘Assisted Dying in Australia: A Values-Based Model for Reform’ in Ian Freckleton and Kerry Peterson (eds), *Tensions and Traumas in Health Law* (Federation Press 2017) 479, 482.

60 Magnusson (n 41) 31.

61 Willmott and others (n 57) 4; Magnusson (n 41) 9.

62 Roy Morgan is an Australian market research and public opinion polling company. The Morgan Poll is a political poll designed to analyse voting intentions.

63 See the summary of Morgan Poll results in South Australian Voluntary Euthanasia Society, Public Opinion Polls on Voluntary Euthanasia Law Reform in Australia, South Australian Euthanasia Society (SAVES) October 2010 reprinted in Justin Healey (ed) *The Euthanasia Debate* (Spinney Press 2013) 9.

64 Lindy Willmott and Ben White, ‘The Challenging Path to Voluntary Assisted Dying Law Reform in Australia-Victoria as a Successful Case Study’ in Ben White and Lindy Willmott (eds), *International Perspectives on End-of-Life Law Reform: Politics, Persuasion and Persistence* (Cambridge University Press 2021) 84, 86.

65 McGee and others (n 58) 1376.

66 Willmott and White (n 64).

67 Robert Ho and Ronald Penney, ‘Euthanasia and Abortion: Personality Correlates for the Decision to Terminate Life’ (1992) 132 J Soc Psychol 77.

68 McGee and others (n 58) 1368.

69 ibid 1376.

70 ibid 1374.

71 ibid.

72 The Northern Territory, Queensland, Tasmania, Western Australia, and the Australian Capital Territory.

73 New South Wales, Victoria, South Australia.

74 See for example, *Crimes Act 1900* (ACT) s 16; *Crimes Act 1900* (NSW) s 31A; *Criminal Law Consolidation Act 1935* (SA) s 13A (1); *Crimes Act 1958* (Vic) s 6A.

75
*Crimes Act 1900* (ACT) s 17; *Crimes Act 1900* (NSW) s 31C; *Criminal Code 1983* (NT) s 162; *Criminal Code 1899* (Qld) s 311; *Criminal Law Consolidation Act 1935* (SA) s 13A(5); *Criminal Code Act 1924* (Tas) 163; *Crimes Act 1958* (Vic) s 6B(2); *Criminal Code 1913* (WA) s 288.

76 Except for those jurisdictions where VAD laws are in operation. This is discussed in detail below.

77
*Crimes Act 1900* (ACT) s 12; *Crimes Act 1900* (NSW) s 18; *Criminal Code 1983* (NT) s 156; *Criminal Code 1899* (Qld) ss 302, 305; *Criminal Law Consolidation Act**1935* (SA) s 11; *Criminal Code Act 1924* (Tas) ss 156, 158; *Crimes Act 1958* (Vic) s 3; *Criminal Code 1913* (WA) s 279. Common law also applies to this offence in NSW, SA, and Vic.

78
*Crimes Act 1900* (ACT) s 15; *Crimes Act 1900* (NSW) s 18; *Criminal Code 1983* (NT) s 160; *Criminal Code 1899* (Qld) s 303; *Criminal Law Consolidation Act 1935* (SA) s 13; *Criminal Code Act 1924* (Tas) 159; *Crimes Act 1958* (Vic) s 421(1); *Criminal Code 1913* (WA) s 280. Common law also applies to this offence in NSW, SA, and Vic.

79 Victoria is Australia’s second smallest state and has over 6 million residents with a median residential age of 38 years. Around 4.9 million live in greater capital area; see <https://www.abs.gov.au/articles/snapshot-vic-2021> (last accessed 6 September 2023).

80 Margaret O’Connor and others, ‘Documenting the Process of Developing the Victorian Voluntary Assisted Dying Legislation’ (2018) 42 Aust Health Rev 621, 622.

81 Courtney Hempton and Catherine Mills, ‘Constitution of “The Already Dying”: The Emergence of Voluntary Assisted Dying in Victoria’ (2021) 18 Bioeth Inq 265, 275.

82 Western Australia, geographically Australia’s largest State, has around 2.7 million residents whose median age is 38 years. Around 2,1 million live in the capital city Perth; see <https://www.abs.gov.au/articles/snapshot-wa-2021> (last accessed 6 September 2023).

83 Tasmania, the geographically smallest Australian State, has 558,000 residents with a median age of 38 years. Around 309,000 residents live outside the capital city area; see <https://www.abs.gov.au/articles/snapshot-tas-2021> (last accessed 6 September 2023).

84 South Australia, Australia’s fourth largest State, has 1.8 million residents with a median age of 38 years. Around 1.4 million live in the greater capital area; see <https://www.abs.gov.au/articles/snapshot-sa-2021> (last accessed 6 September 2023).

85 Queensland, Australia’s second largest State, has 5.2 million people with a median age of 38 years. Over half the population lives outside of the capital city area; see <https://www.abs.gov.au/articles/snapshot-qld-2021> (last accessed 6 September 2023).

86 New South Wales, Australia’s third smallest State, has a population of around 8.1 million with a median age of 38 years. Around 5.2 million residents live in the capital city area; see <https://www.abs.gov.au/articles/snapshot-nsw-2021> (last accessed 6 September 2023).

87 Vic VAD Act s 9(1)(d); WA VAD Act s 16(1)(c); Tas VAD Act s 6(1); SA VAD Act s 26(1)(d); NSW VAD Act s 16(1)(d)(ii)(B). In Victoria, Tasmania, and South Australia the illness, disease, or condition must be incurable. In Western Australia and New South Wales, it is required that the disease will, on the balance of probabilities, cause death. In Queensland, the disease does not have to be incurable but must cause death, Qld VAD Act s 10(1)(a)(i).

88 Vic VAD Act ss 9(1)(d)(iii), 9(4); WA VAD Act s 16(1)(c)(ii); Tas VAD Act s 6(1)(c); SA VAD Act ss 26(1)(d)(iii), 26 (4); NSW VAD Act s 16(1)(d).

89 Qld VAD Act s 10(1)(a)(ii).

90 Vic VAD Act s 9(1)(d); WA VAD Act s 16(1)(c); Tas VAD Act s 6(1); SA VAD ACt s 26(1)(d); Qld VAD Act s 10(1); NSW VAD Act s 16(1)(d)(iii).

91 Vic VAD Act s 9(1)(b); WA VAD Act s 16(1)(b); TAS VAD Act ss 10(1)(b), 11 (in Tasmania it also suffices that where a person is not a permanent resident of Australia the person has been resident in Australia for at least 3 continuous years immediately before the person makes the relevant first request); SA VAD Act s 26(1)(b); Qld VAD Act s 10(1)(e) (in Queensland it also suffices that a person who is not a permanent resident of Australia has been ordinarily resident in Australia for at least 3 years immediately before the person makes the first request. In addition, exemptions from Australian residency can be granted by the chief executive under certain circumstances); NSW VAD Act s 16(1)(b),(c) (in NSW it also suffices that a person who is not a permanent resident of Australia has been ordinarily resident in Australia for at least 3 years immediately before the person makes the first request; exemptions to the residency requirements can be granted; NSW VAD Act s 17).

92 Vic VAD Act s 9(1)(a); WA VAD Act s 16(1)(a); Tas VAD Act s 10(1)(a); SA VAD Act s 26(1)(a); Qld VAD Act s 10(1)(d); NSW VAD Act s 16(1)(a).

93 Vic VAD Act ss 9(1)(c), 20(1)(a), (c)–(d), 29(1)(a), (c)–(d); WA VAD Act ss 6(2), 16(1)(d), (f); Tas VAD Act ss 12, 13, 53; 1 SA VAD Act s 26(1)(c); Qld VAD Act s 10(1)(b); NSW VAD Act s 16(1)(f),(g),(h). That the request is enduring is per se no eligibility criterion in Victoria, Tasmania, South Australia, and Queensland. However, in Victoria and South Australia the practitioners must each be satisfied that the person’s request ‘is enduring’ in the context of their eligibility assessment: Vic VAD Act ss 20(1)(d); 29(1)(d); SA VAD Act ss 38(1)(d); 47(1)(d). In contrast, in Western Australia and New South Wales this is one of the eligibility criteria proper: WA VAD Act s 16(1)(f); NSW VAD Act s 16(1)(h). The term ‘enduring’ does not feature in the Tas or Qld VAD Acts.

94 Magnusson (n 41) 31.

95 See the summary of Morgan Poll results in South Australian Voluntary Euthanasia Society (n 63).

96 Clare Blumer, ‘Vote Compass: Aussies Want It, but Euthanasia Still a “Great Untouched Issue”’ *ABC News* (26 May 2016) <https://www.abc.net.au/news/2016-05-25/vote-compass-euthanasia/7441176> (last accessed 9 September 2023).

97 Willmott and White (n 64) 102.

98 ibid.

99 O’Connor and others (n 80) 624.

100 Victoria, Parliamentary Debates, Legislative Assembly, 18 October 2017, 3229 (David Morris).

101 Duckett (n 49) 397.

102 ibid.

103 ibid.

104 Victoria, Parliamentary Debates, Legislative Assembly 17 October 2017, 3063 (Samuel Hibbins) <https://www.parliament.vic.gov.au/images/stories/daily-hansard/Assembly_2017/Assembly_Daily_Extract_Tuesday_17_October_2017_from_Book_13.pdf> (last accessed 9 September 2023).

105 Victoria, Parliamentary Debates, Legislative Council, 3 November 2017, 5730 (Inga Peulich) criticising that the process had been ‘hijacked’ and driven by the government and supporting minor parties; <www.parliament.vic.gov.au/images/stories/daily-hansard/Council_2017/Council_Daily_Extract_Friday_3_November_2017_from_Book_18.pdf> (last accessed 9 September 2023).

106 Hibbins (n 104).

107 Parliament Joint Select Committee on End of Life Choices, ‘My Life, My Choice The Report of the Joint Select Committee on End of Life Choices’ (Western Australia, 2018) 197 <https://www.parliament.wa.gov.au/Parliament/commit.nsf/(Report+Lookup+by+Com+ID)/71C9AFECD0FAEE6E482582F200037B37/$file/Joint%20Select%20Committe%20on%20the%20End%20of%20Life%20Choices%20-%20Report%20for%20Website.pdf> (last accessed 9 September 2023).

108 Duckett (n 49) 398.

109 Ben White and others, ‘International Perspectives on Reforming End-of-Life Law’ in Ben White and Lindy Willmott (eds), *International Perspectives on End-of-Life Law Reform: Politics, Persuasion and Persistence* (Cambridge University Press 2021) 250, 256.

110 Willmott and White (n 64) 102 noting that the process was ‘well-supported by a secretariat which included research officers’ and that ‘Members of the Parliamentary Committee also had an opportunity to travel across Victoria as well as internationally to jurisdictions where VAD is lawful’.

111 White and others (n 109).

112 Legal and Social Issues Committee, ‘Inquiry into End of Life Choices: Final Report’ (Victoria 2016) xiii.

113 ibid 4.

114 This included two Canadian jurisdictions, The Netherlands, one US jurisdiction and Switzerland. See also O’Connor and others (n 80) 623.

115 Legal and Social Issues Committee (n 112) 8.64.

116 Hibbins (n 104) V 3062–3063.

117 Ministerial Advisory Panel on Voluntary Assisted Dying, ‘Voluntary Assisted Dying Bill: Discussion Paper’ (Victoria 2017).

118 ibid 72.

119 Western Australian Government, ‘Response Joint Select Committee on End-of-Life Choices Report My Life, My Choice’ <https://www.health.wa.gov.au/~/media/Files/Corporate/Reports-and-publications/End-of-life/End-of-Life-My-Life.pdf> (last accessed 9 September 2023).

120 Western Australian Parliament, ‘Joint Select Committee on End of Life Choices’ Report 1 My Life, My Choice’ (Western Australia 2018) <https://www.parliament.wa.gov.au/parliament/commit.nsf/(Report+Lookup+by+Com+ID)/71C9AFECD0FAEE6E482582F200037B37/$file/Joint+Select+Committe+on+the+End+of+Life+Choices+-+Report+for+Website.pdf> (last accessed 9 September 2023).

121 Department of Health, ‘Ministerial Expert Panel on Voluntary Assisted Dying: Final Report’ (Western Australia 2019) 39 <https://ww2.health.wa.gov.au/~/media/Files/Corporate/general-documents/Voluntary-assisted-dying/PDF/voluntary-assisted-dying-final-report.pdf> (last accessed 9 September 2023).

122 Department of Health, ‘Ministerial Expert Panel on Voluntary Assisted Dying: Final Report’ (Western Australia 2019).

123 Duckett (n 49) 394.

124 For more information on Dying with Dignity Victoria see <https://www.dwdv.org.au/about-us/history/>.

125 See for example: Dying with Dignity Australia, ‘Submission to the Victorian Government Legal and Social Issues Committee Inquiry into End of Life Choices (July 2015) <https://www.dwdv.org.au/wp-content/uploads/2020/08/Submission_625_-_Dying_with_Dignity_Victoria_150730.pdf> (last accessed 6 September 2023).

126 For more information on Go Gentle Australia see <https://www.gogentleaustralia.org.au/about> (last accessed 6 September 2023).

127 For more information on the podcast see <https://www.wheelercentre.com/wlr-articles/better-off-dead/>.

128 Peter Fitz Simons, ‘Opinion: The Vulnerable v the Entitled: How VAD Has Become a “Hugely Unfair Fight”’ *The Sydney Morning Herald* (15 May 2022).

129 Go Gentle Australia, ‘Voluntary Assisted Dying Bill—Discussion Paper Response from Go Gentle Australia’ (6 April 2017) <https://assets.nationbuilder.com/gogentleaustralia/pages/2605/attachments/original/1671592552/2017-04_GGA_response_to_VIC_VAD_Bill_Discussion_Paper.pdf?1671592552> (last accessed 6 September 2023).

130 Andrew Denton, ‘Opinion: The Dying Deserve a Compassionate Choice when Palliative Care Fails’ *The Australian* (14 July 2017).

131 Andrew Denton, ‘Opinion: Euthanasia Move is Conservative but Correct’ *The Sydney Morning Herald* (8 December 2016); Andrew Denton, ‘Comment: Why Are We So Scared of Euthanasia?’ *The Sydney Morning Herald* (23 February 2016).

132 Victoria Laurie, ‘Knowing When It’s Time to Go (2016, Sep 29) *The Australian* (29 September 2016); Greg Brown, ‘(2016, Sep 23) Battle for Right to Die Revived’ 20 Years On’ *The Australian* (23 September 2016).

133 Gay Alcorn and Melissa Davey, ‘Victorians Overwhelmingly Support Voluntary Euthanasia, Survey Finds’ *The Guardian* (21 September 2017) <https://www.theguardian.com/society/2017/sep/21/victorians-overwhelmingly-support-voluntary-euthanasia-survey-finds> (last accessed 6 September 2023); Benjamin Preiss and Noel Towell, ‘MPs Push for Six-Month Life Expectancy to Qualify for Voluntary Euthanasia Laws’ *The Age* (1 November 2017) <https://www.theage.com.au/national/victoria/mps-push-for-sixmonth-life-expectancy-to-qualify-for-voluntary-euthanasia-laws-20171101-gzcvwo.html> (last accessed 6 September 2023).

134 Victoria, Parliamentary Debates, Legislative Council, Parliamentary Debates 2 November 2017, 5575 (Colleen Hartland) <https://www.parliament.vic.gov.au/images/stories/daily-hansard/Council_2017/Council_Daily_Extract_Thursday_2_November_2017_from_Book_18.pdf> (last accessed 6 September 2023).

135 Victoria, Parliamentary Debates, Legislative Council, 2 November 2017, 5691 (Sue Pennicuik) <https://www.parliament.vic.gov.au/images/stories/daily-hansard/Council_2017/Council_Daily_Extract_Thursday_2_November_2017_from_Book_18.pdf> (last accessed 6 September 2023).

136 Victoria, Parliamentary Debates, Legislative Council, 2 November 2017, 5687 (the Hon. Gayle Tierney) <https://www.parliament.vic.gov.au/images/stories/daily-hansard/Council_2017/Council_Daily_Extract_Thursday_2_November_2017_from_Book_18.pdf> (last accessed 6 September 2023).

137 Victoria, Parliamentary Debates, Legislative Assembly, 18 October 2017, 3233 (Stephanie Ryan) <https://www.parliament.vic.gov.au/images/stories/daily-hansard/Assembly_2017/Assembly_Daily_Extract_Wednesday_18_October_2017_from_Book_13.pdf> (last accessed 6 September 2023).

138 Hibbins (n 104) 3063.

139 Western Australia, Parliamentary Debates, Legislative Council, 16 October 2019, 7763 (the Hon Colin de Grussa); Western Australia, Parliamentary Debates, Legislative Council, 31 October 2019, 8671 (the Hon. Nick Goiran) <https://www.parliament.wa.gov.au/Hansard/Hansard.nsf/0/2CF6C51F120D15B3482584A8001DD671/$file/C40%20S1%2020191031%20All.pdf> (last accessed 6 September 2023).

140 Western Australia, Parliamentary Debates, Legislative Council, 16 October 2019, 7751 (the Hon. Martin Aldridge) <https://www.parliament.wa.gov.au/Hansard/Hansard.nsf/0/65AC08BE5D30F42F48258497002AF24B/$file/C40%20S1%2020191016%20All.pdf> (last accessed 6 September 2023); Western Australia, Parliamentary Debates, Legislative Council, 17 October 2019, 7854 (the Hon Colin de Grussa) <https://www.parliament.wa.gov.au/Hansard/Hansard.nsf/0/542E1024AF021A15482584B000253EB9/$file/C40%20S1%2020191017%20All.pdf> (last accessed 6 September 2023).

141 Western Australia, Parliamentary Debates, Legislative Council, 5 December 2019, 9895 (the Hon Alannah MacTiernan) <https://www.parliament.wa.gov.au/Hansard/Hansard.nsf/0/0A88CFD7496821FC482584CB001B1BA9/$file/C40%20S1%2020191205%20All.pdf> (last accessed 6 September 2023).

142 See Willmott and White (n 64) 102 suggesting that ‘such an extensive reform process’ as took place in the first two States may not necessarily be required to bring about law reform in other jurisdictions.

143 Tasmanian Policy Exchange at the University of Tasmania, *Independent Review of the End of Life Choices (Voluntary Assisted Dying) Bill 202*0 (University of Tasmania 2021) <https://www.utas.edu.au/__data/assets/pdf_file/0009/1475568/Independent-review-voluntary-assisted-dying.pdf> (last accessed 6 September 2023).

144 Tasmania, Parliamentary Debates, House of Assembly, 10 November 2020 (Cassy O’Connor) 3 <https://search.parliament.tas.gov.au/Search/search/search?IW_FIELD_ADVANCE_PHRASE=Tuesday%2010%20November%202020&IW_DATABASE=Hansard&IW_SORT=-9> (last accessed 6 September 2023).

145 Tasmania, Parliamentary Debate, House of Assembly, 3 December 2020 (resumed 4 December 2020), 123 (Alison Standen) <https://search.parliament.tas.gov.au/Search/search/search?IW_FIELD_ADVANCE_PHRASE=Thursday%203%20December%202020&IW_DATABASE=Hansard&IW_SORT=-9> (last accessed 6 September 2023)

146 Tasmania, Parliamentary Debates, House of Assembly, 3 December 2020 (resumed 4 December 2020), 152 (Jenna Butler) <https://search.parliament.tas.gov.au/Search/search/search?IW_FIELD_ADVANCE_PHRASE=Thursday%203%20December%202020&IW_DATABASE=Hansard&IW_SORT=-9> (last accessed 6 September 2023).

147 South Australia, Parliamentary Debates, House of Assembly, 26 May 2021, 5981 (the Hon SS Marshall) <https://hansardsearch.parliament.sa.gov.au/daily/lh/2021-05-26/55?sid=39b543c9f3f54621bf> (last accessed 6 September 2023).

148 See Queensland Parliament Health, Communities, Disability Services and Domestic and Family Violence Prevention Committee, ‘Voluntary Assisted Dying—Report 34’ (Queensland 2020).

149 Queensland Law Reform Commission, ‘A Legal Framework for Voluntary Assisted Dying Report No 79’ (Queensland 2021) <https://www.qlrc.qld.gov.au/__data/assets/pdf_file/0020/681131/qlrc-report-79-a-legal-framework-for-voluntary-assisted-dying.pdf> (last accessed 6 September 2023).

150 Queensland, Parliamentary Debates, 14 September 2021 (Hon. SJ Miles) 2553 <https://documents.parliament.qld.gov.au/events/han/2021/2021_09_14_WEEKLY.pdf> (last accessed 6 September 2023).

151 However, there was a parliamentary inquiry into the terms of a Bill which had already been tabled. This did not lead to specific recommendations for law reform. New South Wales Parliament Legislative Council, Standing Committee on Law and Justice, ‘Report no. 79. Voluntary Assisted Dying Bill 2021’ (February 2022) <www.parliament.nsw.gov.au/lcdocs/inquiries/2838/Report%20No%2079%20-%20Voluntary%20Assisted%20Dying%20Bill%202021.pdf> (last accessed 6 September 2023).

152 New South Wales, Legislative Council, Voluntary Assisted Dying Bill 2021, Second Reading Speech, 23 February 2022 (The Hon. Adam Searle) <https://www.parliament.nsw.gov.au/Hansard/Pages/HansardResult.aspx#/docid/’HANSARD-1820781676-88102’> (last accessed 6 September 2023).

153 ibid.

154 See Willmott and others (n 57) 9-10.

155 Markus Mannheim, ‘Federal Parliament Overturns 25-Year-Old Ban on Euthanasia Laws in ACT and Northern Territory’ *ABC News* (1 December 2022) <https://www.abc.net.au/news/2022-12-01/parliament-lifts-ban-on-territory-euthanasia-laws/101692028> (last accessed 6 September 2023).

156 Aimee Woodmass, ‘Australia Territories Free to Make Law on Voluntary Assisted Dying After Senate Vote’ *Jurist* (2 December 2022) <https://www.jurist.org/news/2022/12/australian-territories-free-to-make-law-on-voluntary-assisted-dying-after-senate-passes-new-bill/> (last accessed 6 September 2023).

157 Margaret Somerville, ‘Legalising Euthanasia: Why Now?’ (1996) 68(3) Aust Quart 1, 4.

158 ibid.

159 Kerridge and Mitchell (n 54)278.

160 White and others (n 109) 263.

161 ibid.

162 ibid 261-262.

163 Voluntary Assisted Dying Review Board, Report of Operations July 2021 to June 2022 (Victoria 2022) 1 <https://www.safercare.vic.gov.au/sites/default/files/2022-09/Voluntary%20Assisted%20Dying%20Review%20Board%20Report%20of%20Operations%20July%202021-June%2022_FINAL.pdf> (last accessed 6 September 2023).

164 This is a significant increase from 190 total deaths between 2019–2020 and 310 total deaths between 2020–2021. ibid 5.

165 ibid 25.

166 ibid.

167 Voluntary Assisted Dying Review Board Western Australia, Annual Report 2021–2022, 16 November 2022 <https://ww2.health.wa.gov.au/~/media/Corp/Documents/Health-for/Voluntary-assisted-dying/VAD-Board-Annual-Report-2021-22.pdf> (last accessed 6 September 2023).

168 ibid 24.

169 ibid 35.

170 ibid 4.

171 Voluntary Assisted Dying Commission Tasmania, Voluntary Assisted Dying Update (10 March 2023) <https://www.health.tas.gov.au/news/articles/voluntary-assisted-dying-update> (last accessed 6 September 2023).

172 White and Willmott (n 58) 617.

173 ibid 619.

